# Puerperal Septicæmia (Parturient Apoplexy, Milk-fever, Etc.)1Read before the Western Ontario Veterinary Association, December 29, 1897.

**Published:** 1898-07

**Authors:** J. L. Clark

**Affiliations:** Stratford, Ont.


### PUERPERAL SEPTICÆMIA (PARTURIENT APOPLEXY, MILK-FEVER, ETC.).[1]

BY J. L. CLARK, V.S.,
STRATFORD, ONT.

THERE is no subject in the whole range of veterinary practice that has given rise to so much discussion or is so important as that which we now propose to discuss. The various terms that have from time to time been applied to the disease—parturient apoplexy, milk-fever, etc.—indicate to some extent the views held regarding its pathology. The term puerperal septicæmia, though familiar to all practitioners, so far as I know, has never been applied to the condition familiarly known as parturient apoplexy, and I believe as the pathology of the disease becomes recognized the term will be approved by all veterinarians.

Septicæmia is caused by the entrance into the blood of a poison absorbed from a wound the discharge of which is putrid. Pathologists recognize two distinct forms of the disease: (*a*) Septic intoxication, or sapræmia, in which there is absorbed into the blood the chemical products or ptomaïns of putrefaction. The quantity of toxin does not increase after absorption, so that the effects are proportional to the amount absorbed. (*b*) Septic infection, in which there is in the blood a specific virus which increases in the system, producing its toxins there, so that, unlike septic intoxication, its effects are not proportional to the original dose.

Bacteriologists have shown that the process of putrefaction is a fermentation dependent upon the presence in dead animal matter of pathogenic microörganisms. There are various forms—round,

[1] Read before the Western Ontario Veterinary Association, December 29, 1897.

oval, rod-shaped, cylindrical, spiral, etc. Those giving rise to septicæmia belong to the class called micrococci, and are of two varieties, called streptococcus pyogenes aureus and staphylococcus pyogenes aureus.

The various conditions, viz., plethora, heavy milkers, debility, season, etc., that are given as causes of the disease, we regard only as predisposing causes, believing the true causation to be infection of the uterus or any part of the parturient canal by the microörganisms already mentioned. The condition of the uterus and genital tract after delivery presents conditions favorable for the absorption of septic material. In the uterus there is the site of placental separation between the cotyledons and placenta, through which absorption may readily occur. Again, inefficient uterine contractions from any cause, prolonged labor, plethora, debility, etc., lead to retention of portions of fœtal membranes, fœtal matter, muco-secretion, blood-clots; these furnish ample material for the growth of bacteria. Putrefaction results, ptomaïns are absorbed, and the animal is afflicted with puerperal septicæmia.

*Morbid Anatomy.* Blood, dark in color, and may be imperfectly coagulated. Heart flabby; small extravasations of blood beneath pericardium. Peritoneum, pleura, liver, and kidneys swollen and full of blood. Spleen swollen and soft. Mucous membranes of alimentary canal intensely congested; the red blood-corpuscles disintegrated, thereby causing the dark blood and the tissues and vessel-walls to be darkly stained by liberated hæmoglobin. The petechiæ found beneath the serous membranes are due to the capillaries being blocked by clumps of corpuscles. The viscera are congested with venous blood due to feeble cardiac action. Uterus soft, swollen, and imperfectly contracted. Decomposing organic matter present in uterus, and often severe endometritis present.

*Symptoms.* The symptoms are familiar to all: Restlessness, paddling with the hind feet, rumination ceases, secretion of milk stops, paralysis of posterior extremities, sometimes coma, sometimes delirium, contraction of muscles of neck, etc., rapid respiration, high pulse, elevation of temperature. These symptoms all arise from the absorption of the virus from the site of infection, and their severity depends upon the amount absorbed and the resistance offered by the animal tissues to the invading organisms and their chemical products.

*Diagnosis.* It is impossible, without examination of the blood, to distinguish between septic intoxication and septic infection, as the symptoms presented by the two affections are identical. To diagnose septic infection a culture from the blood must be made and the streptococci recognized by microscopic examination.

*Treatment.* Thoroughly clean out uterus by hand, removing all decomposing matter; inject uterus with a solution of carbolic, creolin, or hyd. perchlor. (1 : 1000 or 2 : 1000). Relieve bowels by injections where restlessness, with muscular contraction, is present. Give antispasmodics, of which I have found chlor. hydrate, pot. brom. with spts. ammonia aramat., as a mild diffusible stimulant, most useful. Where heart is weak, digitalis, fl. ext., alcohol and liq. strych. are indicated.

In early stage, where pulse is full and bounding, I recommend a copious abstraction of blood, which relieves the congested organs and diminishes the toxins in the circulation and acts as a cardiac stimulant. In early stages I can also recommend the following: 1st, Aconite, fl. ext., ʒj; alcohol, ℥ij; 2d, Belladonna, fl. ext., ʒij; alcohol, ℥ij. Sig. One teaspoonful, alternating every fifteen minutes until, commencing with the 1st, four doses have been given; then, if any improvement, give nux vomica, fl. ext., ♏x., every hour; if no improvement, repeat.

There have been cases recorded where the disease has manifested itself before parturition and after abortion. You will always find the uterus in that flaccid condition accompanying such cases. Where the uterus is feebly contracted after the douche, give ergot.

